# Composite Magnetic Photocatalyst Bi_5_O_7_I/Mn_x_Zn_1−x_Fe_2_O_4_: Hydrothermal-Roasting Preparation and Excellent Photocatalytic Activity

**DOI:** 10.3390/nano9010118

**Published:** 2019-01-18

**Authors:** Hailong Wang, Longjun Xu, Chenglun Liu, Yuan Lu, Qi Feng, Tingzeng Wu, Ruiqi Wang

**Affiliations:** 1 State Key Laboratory of Coal Mine Disaster Dynamics and Control, Chongqing University, Chongqing 400044, China; cqyywhl@163.com (H.W.); xlclj@cqu.edu.cn (C.L.); cquluyuan@126.com (Y.L.); 18223208007@163.com (Q.F.); tingzengyeh@163.com (T.W.); HYQWRO1314@163.com (R.W.); 2 College of Chemistry and Chemical Engineering, Chongqing University, Chongqing 400044, China

**Keywords:** magnetic photocatalyst, Bi_5_O_7_I/Mn_x_Zn_1−x_Fe_2_O_4_, hydrothermal-roasting method

## Abstract

A new composite magnetic photocatalyst, Bi_5_O_7_I/Mn_x_Zn_1−x_Fe_2_O_4_, prepared by a hydrothermal-roasting method was studied. The photocatalytic properties of Bi_5_O_7_I/Mn_x_Zn_1−x_Fe_2_O_4_ were evaluated by degradation of Rhodamine B (RhB) under simulated sunlight irradiation, and the structures and properties were characterized by X-ray diffraction (XRD), Fourier-transform infrared spectroscopy (FTIR), scanning electron microscopy (SEM), transmission electron microscopy (TEM), ultraviolet-visible light (UV-Vis) diffuse reflectance spectra (DRS), and a vibrating sample magnetometer (VSM). The results indicated that Bi_5_O_7_I/Mn_x_Zn_1−x_Fe_2_O_4_ was an orthorhombic crystal, which was similar to that observed for Bi_5_O_7_I. Bi_5_O_7_I/Mn_x_Zn_1−x_Fe_2_O_4_ consisted of irregularly shaped nanosheets that were 40–60 nm thick. The most probable pore size was 24.1 nm and the specific surface area was 7.07 m^2^/g. Bi_5_O_7_I/Mn_x_Zn_1−x_Fe_2_O_4_ could absorb both ultraviolet and visible light, and the energy gap value was 3.22 eV. The saturation magnetization, coercivity and residual magnetization of Bi_5_O_7_I/Mn_x_Zn_1−x_Fe_2_O_4_ were 3.9 emu/g, 126.6 Oe, and 0.7 emu/g respectively, which could help Bi_5_O_7_I/Mn_x_Zn_1−x_Fe_2_O_4_ be separated and recycled from wastewater under the action of an external magnetic field. The recycling experiments revealed that the average recovery rate of the photocatalyst was 90.1%, and the photocatalytic activity was still more than 81.1% after five cycles.

## 1. Introduction

In recent years, with the progress of nanotechnology and the application of photocatalytic technology in environmental pollution treatment [1,2,3], lots of semiconducting metal-oxide nanostructures were widely used for water purification due to their great photocatalytic performance [4,5]. Grottrup [6] applied Bi for doping ZnO, which significantly enhanced its ability in the photocatalytic degradation of methylene blue. Huang [7] indicated that the rate constant of degradation of 17 β-estradiol over N-doped Bi_2_O_3_ was 6.3 times that obtained over Bi_2_O_3_. Oppong [8] pointed out that the reason for the better photocatalytic performance of Gd–TiO_2_–graphene oxide (GO) nanocomposites compared to pure TiO_2_ was because GO sheets and Gd^3+^ ions are excellent co-catalysts and their presence promotes the reaction sites. Currently, bismuth-based nanometer semiconductors are one of the research hotspots in the field of photocatalytic materials due to their unique electronic structure and excellent absorption ability of ultraviolet and visible light [9,10,11,12]. Bi_5_O_7_I, an oxygen-rich bismuth-based nanometer semiconductor [13,14,15], is composed of a Bi 6*p* orbital at the bottom of the conduction band and Bi 6*s*, O 2*p*, and I 5*p* orbitals at the top of the valence band [16,17]. The Bi 6*s* and O 2*p* orbitals can form several dispersed hybrid valence bands, while the I 5*p* orbital disperses the valence bands further [18,19]. Consequently, the above results accelerate the migration of photo-generated holes and promote the occurrence of oxidation reactions [20,21]. Sun et al. thought that the Bi_5_O_7_ and I sections form a unique hierarchical structure successively along the c-axis orderly. As an accelerator for the separation of photo-generated electron–hole pairs, the permanent electrostatic field between the layers can improve the photocatalytic activity of Bi_5_O_7_I [22]. Xia et al. prepared sheets of Bi_5_O_7_I using the calcining method. However, the photocatalytic degradation rate of Bi_5_O_7_I (0.1 g) for Rhodamine B (RhB) solution (100 mL, 10mg/L) was only 52% in 120 min under simulated sunlight irradiation [23]. In order to enhance the photocatalytic activity of Bi_5_O_7_I, some means were mentioned, such as doping and compounding [24,25,26,27]. The photocatalytic degradation rate of Eu (3%)/Bi_5_O_7_I microspheres was 2.8 times that of Bi_5_O_7_I [28], and the photocatalytic degradation efficiency of a graphitic carbon nitride (g-C_3_N_4_) (10%)–Bi_5_O_7_I nanocomposite was 1.4 times that of Bi_5_O_7_I [29]. 

Most organic pollutants in wastewater can be degraded using photocatalytic technology, with good degradation effects and no secondary pollution. Nevertheless, the complex process, large energy consumption, and low recovery rate are the main disadvantages of the common recovery method, because photocatalytic materials disperse in wastewater uniformly [30]. Mn_x_Zn_1−x_Fe_2_O_4_ is a soft magnetic ferrite material with its own benefits, such as high saturation magnetization, high permeability, low coercive force, low loss, strong stability, and so on [31]. Therefore, composite magnetic photocatalytic materials prepared with Mn_x_Zn_1−x_Fe_2_O_4_ as a magnetic matrix can achieve the goal of magnetic recovery, and the heterojunction formed between Mn_x_Zn_1−x_Fe_2_O_4_ and the photocatalyst is conducive to enhancing the photocatalytic activity of the composite. For instance, Zhang et al. [32] synthesized a Mn_x_Zn_1−x_Fe_2_O_4_/α-Bi_2_O_3_ composite magnetic photocatalyst using the dip-calcination method. However, the recovery rate was only 84.1% under the action of an external magnet, and the degradation time was more than four hours when the degradation rate attained 86.2% after five recoveries. The energy consumption of the preparation process is tremendous, because the precursor of Mn_x_Zn_1−x_Fe_2_O_4_ must be calcined at 1200 °C for three hours. In addition, the synthesis of composite magnetic photocatalytic materials using Bi_5_O_7_I as a photocatalyst and Mn_x_Zn_1−x_Fe_2_O_4_ as the magnetic matrix is rarely reported.

To overcome these shortcomings, a Bi_5_O_7_I/Mn_x_Zn_1−x_Fe_2_O_4_ composite magnetic photocatalyst was prepared using a hydrothermal-roasting method, and the structures and properties were characterized by X-ray diffraction (XRD), Fourier-transform infrared spectroscopy (FTIR), scanning electron microscopy (SEM), transmission electron microscopy (TEM), ultraviolet-visible light (UV-Vis) diffuse reflectance spectra (DRS), and a vibrating sample magnetometer (VSM). In the meantime, the activity and stability of Bi_5_O_7_I/Mn_x_Zn_1−x_Fe_2_O_4_ were evaluated through the degradation of RhB under simulated sunlight irradiation.

## 2. Materials and Methods

Analytical reagents of Bi(NO_3_)_3_·5H_2_O, KI, C_2_H_6_O_2_, Fe_2_(SO_4_)_3_, MnSO_4_·H_2_O, ZnSO_4_·7H_2_O, NaOH, C_28_H_31_ClN_2_O_3_, and HNO_3_ were used as raw materials for sample preparation, and were provided by Chengdu Kelong Chemical Ltd (Chengdu, China).

### 2.1. Preparation of Bi_5_O_7_I/Mn_x_Zn_1−x_Fe_2_O_4_

Mn_x_Zn_1−x_Fe_2_O_4_ was prepared using a hydrothermal method. Firstly, according to the molar ratio of n(MnO):n(ZnO):n(Fe_2_O_3_) = 3.28:1.33:5.39, ZnSO_4_·7H_2_O, MnSO_4_·H_2_O, and Fe_2_(SO_4_)_3_ were dissolved in deionized water. Then, 5 M NaOH aqueous solution was added dropwise to adjust the pH value of the solution to 13 under vigorous stirring. Subsequently, the solution was transferred into a Teflon-lined autoclave for reaction at 200 °C for 5 h. Afterward, the resulting precipitates were washed with deionized water, as well as dilute sulfuric acid, several times, before being dried at 80 °C for 12 h. 

**Scheme 1:**
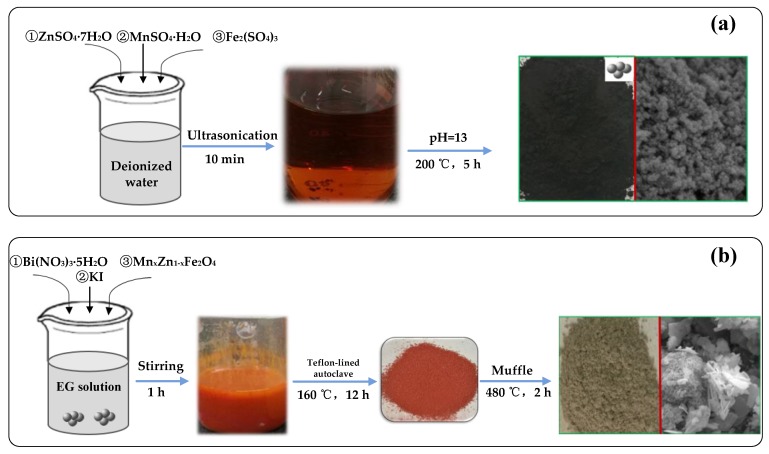
Schematic illustration of the synthetic process for (**a**) Mn_x_Zn_1−x_Fe_2_O_4_ and (**b**) Bi_5_O_7_I/Mn_x_Zn_1−x_Fe_2_O_4._

Bi_5_O_7_I/Mn_x_Zn_1−x_Fe_2_O_4_ was prepared using a hydrothermal-roasting method. Firstly, 5 mL of ethylene glycol (EG) was dissolved in 35 mL of deionized water with stirring for 10 min to gain solution A. Then, 2 mmol Bi (NO_3_)_3_·5H_2_O and 2 mmol KI were continuously dissolved in solution A while stirring to acquire suspension liquid B. Then, 10 wt.% as-prepared Mn_x_Zn_1−x_Fe_2_O_4_ was added into suspension liquid B with continuous stirring for 60 min. Afterward, the mixed turbid solution was transferred into a Teflon-lined autoclave for reaction at 160 °C for 12 h, and the resulting precipitates were washed with deionized water several times. Finally, the precipitates were dried at 80 °C for 5 h and roasted at 480 °C for 2 h in a muffle, obtaining Bi_5_O_7_I/Mn_x_Zn_1−x_Fe_2_O_4_. The synthetic process is displayed in Scheme 1.

### 2.2. Characterization

The structures of the as-prepared products were characterized by X-ray diffraction (XRD; Shimadzu, XRD-6000, Shimadzu, Kyoto, Japan) and Fourier-transform infrared spectroscopy (FTIR; Nicolet iS50, Thermo Fisher Scientific, Waltham, MA, USA). The morphologies and microstructures of the products were observed using scanning electron microscopy (SEM; S4800, Hitachi, Tokyo, Japan) and transmission electron microscopy (TEM; Tecnai G2F20, FEI, Hillsboro, OR, USA). The surfaces and apertures of the products were measured using an automatic multistation surface and aperture analyzer (Quadrasorb 2MP, Quantachrome, Boynton Beach, FL, USA). The element contents in the composite were analyzed by X-ray photoelectron spectroscopy (XPS, ESCALAB250Xi, Thermo Fisher Scientific, Waltham, MA, USA). The optical absorption ability and magnetic performance of the products were identified with UV-Vis diffuse reflectance spectra (UV-Vis DRS; TU1901, Beijing, China) and a vibrating sample magnetometer (VSM; 7410, Lake Shore, Westerville, OH, USA) respectively.

### 2.3. Photocatalytic Evaluation

The photocatalytic properties of the samples were evaluated through the degradation of Rhodamine B (RhB) under simulated sunlight irradiation. Firstly, 0.1 g of photocatalyst and 100 mL of RhB aqueous solution (10 mg·L^−1^) were put into a beaker and stirred for 30 min in the dark to reach the adsorption balance. Then, the mixtures were irradiated with a xenon lamp (CEL-HXF3000, AULTT) of 300 W, and the ultraviolet-visible emission spectrum. Then, 4 mL of the solution was withdrawn at set time intervals, before being centrifuged at 4000 rpm for 5 min to get the supernatant. Finally, the characteristic absorbance of RhB was measured using a UV-Vis spectrophotometer. 

## 3. Results and Discussion

### 3.1. Structure Characteristics

**Figure 1:**
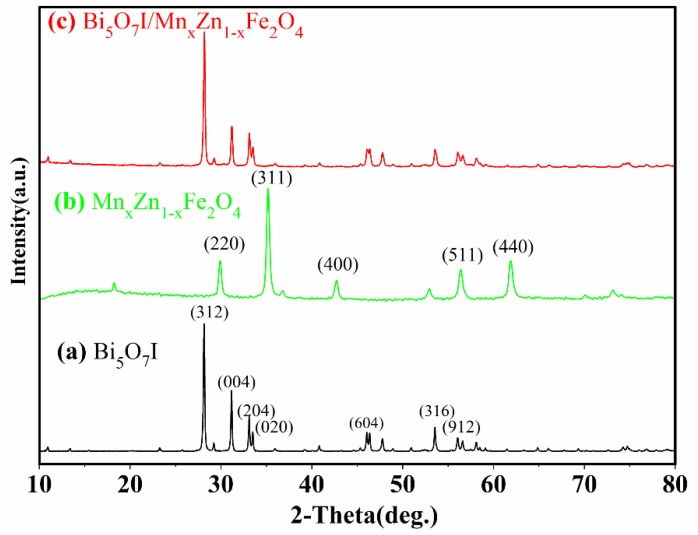
(**a**) X-ray diffraction (XRD) patterns of Bi_5_O_7_I; (**b**) XRD patterns of Mn_x_Zn_1−x_Fe_2_O_4_; (**c**) XRD patterns of Bi_5_O_7_I/Mn_x_Zn_1−x_Fe_2_O_4_.

Figure 1 presents the XRD patterns of Mn_x_Zn_1−x_Fe_2_O_4_, Bi_5_O_7_I, and Bi_5_O_7_I/Mn_x_Zn_1−x_Fe_2_O_4_. As displayed, the diffraction peaks, located at 28.24°, 31.23°, 33.14°, 33.55°, 47.82°, 53.60°, and 56.12°, were assigned to the (312), (004), (204), (020), (224), (316), and (912) planes of orthorhombic Bi_5_O_7_I (JCPDS file No. 40-0538). Neither the preferred growth direction nor the crystal structure of Bi_5_O_7_I were changed by Mn_x_Zn_1−x_Fe_2_O_4_, because the characteristic peaks of Bi_5_O_7_I/Mn_x_Zn_1−x_Fe_2_O_4_ were basically consistent with Bi_5_O_7_I. Interestingly, the peak of the (912) plane of Bi_5_O_7_I/Mn_x_Zn_1−x_Fe_2_O_4_ overlapped with the (511) plane of the Mn_x_Zn_1−x_Fe_2_O_4_. The average crystalline size of Bi_5_O_7_I/Mn_x_Zn_1−x_Fe_2_O_4_ was 71.3 nm, as calculated using the Scherrer formula. The cell parameters were as follows: a = 16.8036 Å, b = 5.0721 Å, c = 11.7316 Å, α = β = γ = 90°.

**Figure 2:**
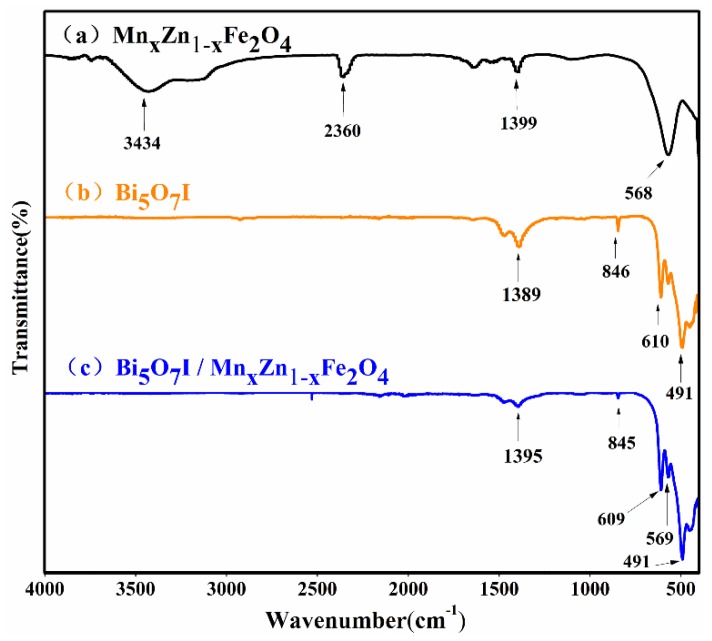
(**a**) Fourier-transform infrared (FTIR) spectrum of Mn_x_Zn_1−x_Fe_2_O_4_; (**b**) FTIR spectrum of Bi_5_O_7_I; (**c**) FTIR spectrum of Bi_5_O_7_I/Mn_x_Zn_1−x_Fe_2_O_4_.

The FTIR spectra of Mn_x_Zn_1−x_Fe_2_O_4_, Bi_5_O_7_I, and Bi_5_O_7_I/Mn_x_Zn_1−x_Fe_2_O_4_ are shown in Figure 2. The characteristic peaks at 3434 cm^−1^ and 2360 cm^−1^ were attributed to the stretching vibration and bending vibration of the hydroxyl group (–OH) from surface-adsorbed water, respectively [33]. Typical Raman bands of the Fe–O–Fe bond and the stretching vibration of the Zn–O bond were located at 1399 cm^−1^ and 568 cm^−1^, respectively [34]. The intensive signals around 1389 cm^−1^, 846 cm^−1^, and 610 cm^−1^ referred to the bending vibration of the Bi–O bond, and 491 cm^−1^ referred to the stretching vibration of the Bi–O bond [35]. Figure 2c indicates that the hydroxyl group in Mn_x_Zn_1−x_Fe_2_O_4_ disappeared because of the roasting process. There was a slight blueshift about the bending vibration of the Bi–O bond (from 610 cm^−1^ to 609 cm^−1^) and the stretching vibration of the Fe–O–Fe bond (from 1399 cm^−1^ to 1395 cm^−1^). Moreover, the stretching vibration of Zn–O bond (569 cm^−1^) could be seen in Bi_5_O_7_I/Mn_x_Zn_1−x_Fe_2_O_4_. 

**Figure 3:**
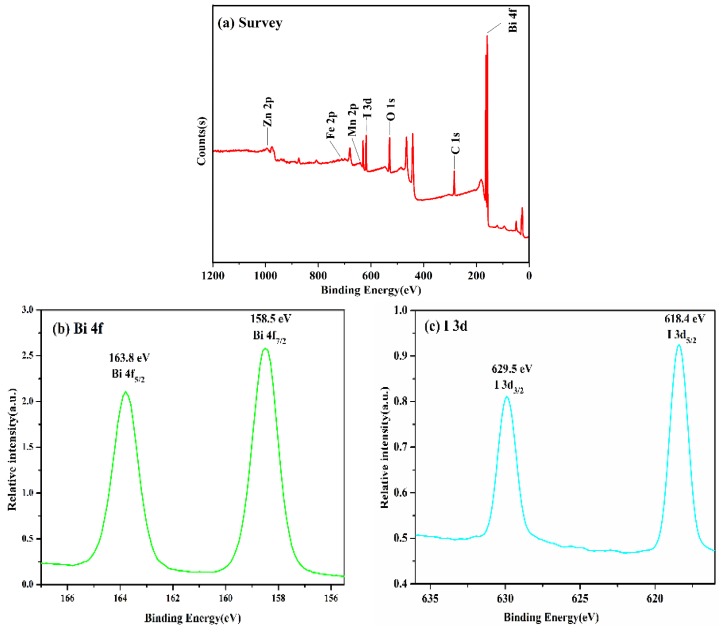

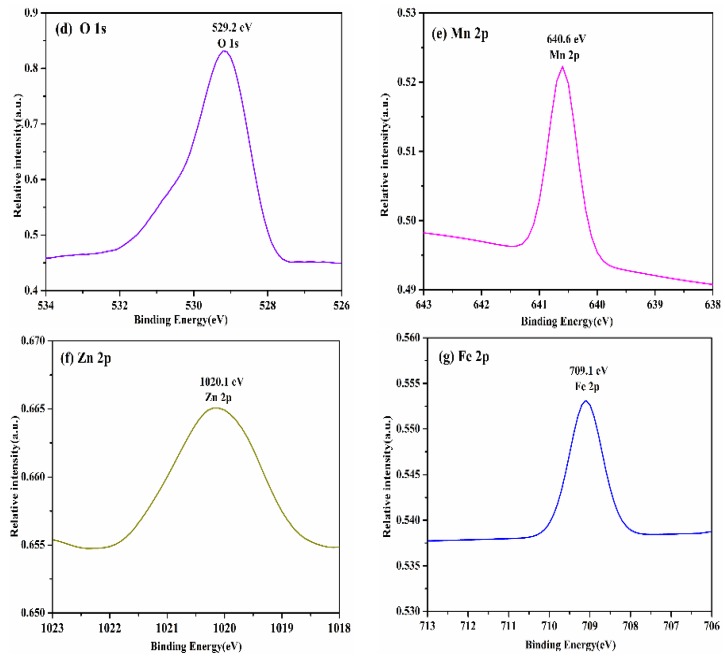
(**a**) X-ray photoelectron spectroscopy (XPS) survey spectra; (**b**–**g**) corresponding high-resolution XPS spectra of each element.

The chemical composition and state of the Bi_5_O_7_I/Mn_x_Zn_1−x_Fe_2_O_4_ sample were investigated using XPS. The survey spectra in Figure 3a reveal that the elements of Bi, I, O, Mn, Zn, and Fe exist in Bi_5_O_7_I/Mn_x_Zn_1−x_Fe_2_O_4_. The atomic percentages of Bi, I, O, Mn, Zn, and Fe were 27.4%, 3.74%, 58.13%, 6.51%, 0.35%, and 3.87%, respectively. Figure 3b describes that the two characteristic peaks of Bi 4*f*_5/2_ and Bi 4*f*_7/2_ were located at 163.8 eV and 158.5 eV in the high-resolution spectra. The I 3*d*_3/2_ and I 3*d*_5/2_ peaks in Figure 3c could be fitted with two peaks at 629.6 eV and 618.4 eV. As can be seen from Figure 3d–g, the signals at 529.2 eV, 640.6 eV, 1020.4eV, and 709.1eV were attributed to the O 1*s*, Mn 2*p*, Zn 2*p*, and Fe 2*p* orbitals, respectively.

**Figure 4:**
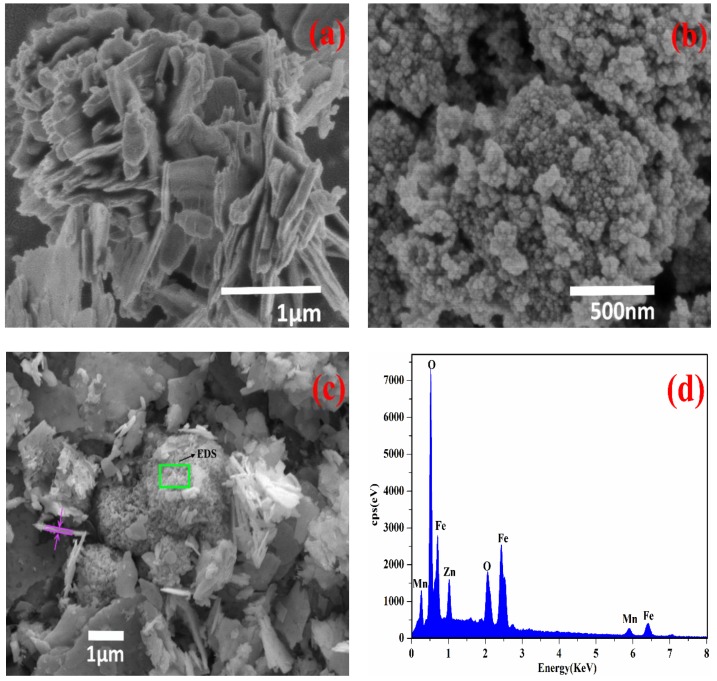
(**a**) SEM image of Bi_5_O_7_I; (**b**) SEM image of Mn_x_Zn_1−x_Fe_2_O_4_; (**c**) SEM image of Bi_5_O_7_I/Mn_x_Zn_1−x_Fe_2_O_4_; (**d**) energy-dispersive X-ray spectroscopy (EDS) spectrum of Mn_x_Zn_1−x_Fe_2_O_4_.

In order to observe the morphology of the materials, the samples were characterized by SEM. As seen from Figure 4, Bi_5_O_7_I was composed of irregularly shaped nanosheets, while Mn_x_Zn_1−x_Fe_2_O_4_ was a spherical particle. The layer thickness of the nanosheets was around 40–60 nm, as shown in Figure 4c. The energy-dispersive X-ray spectroscopy (EDS) spectrum confirmed that the particles in Figure 4c were Mn_x_Zn_1−x_Fe_2_O_4_, demonstrating the successful creation of the Mn_x_Zn_1−x_Fe_2_O_4_ and Bi_5_O_7_I compound.

**Figure 5:**
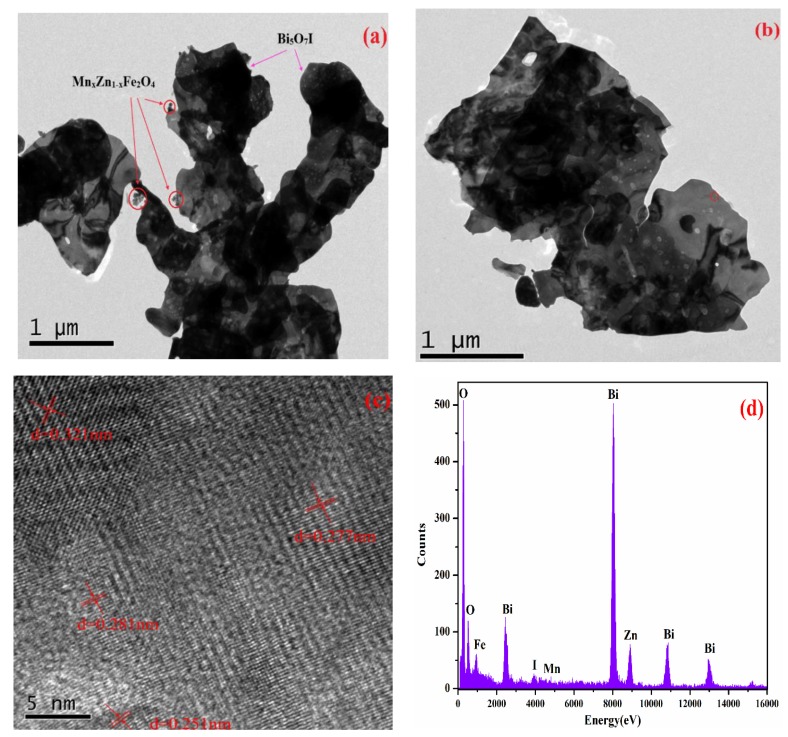
(**a**,**b**) TEM image of Bi_5_O_7_I/Mn_x_Zn_1−x_Fe_2_O_4_; (**c**) high-resolution TEM (HRTEM) image of Bi_5_O_7_I/Mn_x_Zn_1−x_Fe_2_O_4_; (**d**) EDS spectrum of Bi_5_O_7_I/Mn_x_Zn_1−x_Fe_2_O_4_.

Figure 5a,b display that lots of irregular holes on the sample surface existed. At the same time, many particles of Mn_x_Zn_1−x_Fe_2_O_4_ were in contact with the gray shells of Bi_5_O_7_I. The high-resolution TEM (HRTEM) image of the circular region in Figure 5b reveals that Bi_5_O_7_I/Mn_x_Zn_1−x_Fe_2_O_4_ was polycrystalline due to the different orientation of the crystal surface. The three fringe spacings of 0.321 nm, 0.277 nm, and 0.281 nm between neighboring crystal lattices corresponded to the crystal surface (312), (204), and (004) of the Bi_5_O_7_I crystal, and the fringe spacing of 0.251 nm corresponded to the crystal surface (311) of Mn_x_Zn_1−x_Fe_2_O_4_. The EDS result proved the existence of Bi, I, O, Mn, Zn, and Fe elements, consistent with the XPS investigation. 

**Figure 6:**
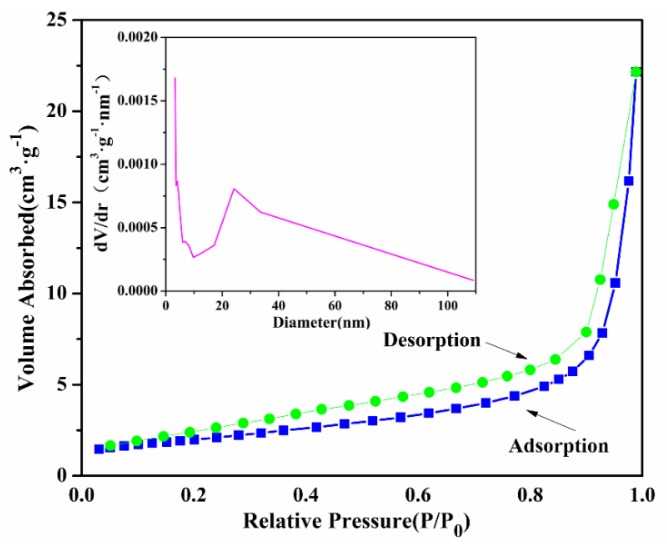
The N_2_ adsorption–desorption isotherm of Bi_5_O_7_I/Mn_x_Zn_1−x_Fe_2_O_4_. The pore diameter distribution curve is shown in the inset image.

A specific surface analyzer was utilized to research the specific surface area and pore diameter distribution of Bi_5_O_7_I/Mn_x_Zn_1−x_Fe_2_O_4_. From Figure 6, according to the Brunauer isotherm classification method, the adsorption–desorption isotherm belonged to class IV. The pore diameter distribution curve described that the most probable pore size of Bi_5_O_7_I/Mn_x_Zn_1−x_Fe_2_O_4_ was 24.1 nm. Furthermore, the specific surface area of the Bi_5_O_7_I/Mn_x_Zn_1−x_Fe_2_O_4_ sample calculated using the Brunauer–Emmett–Teller (BET) model was 7.07 m^2^/g.

### 3.2. Absorption Light Ability and Magnetic Properties

**Figure 7:**
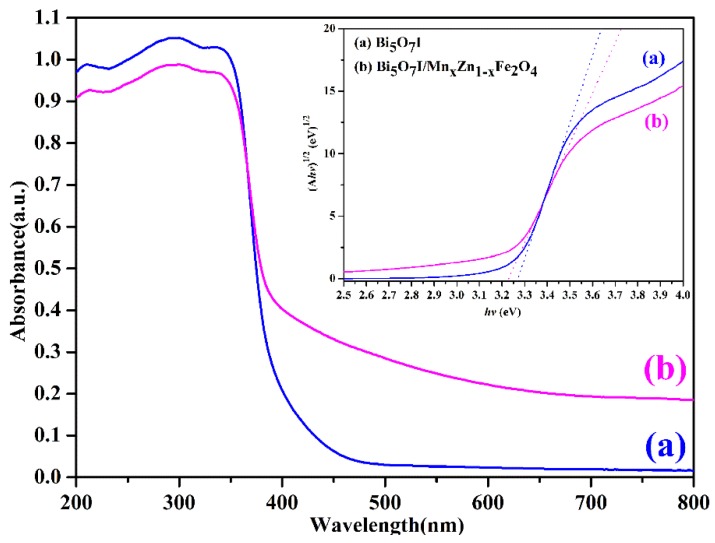
(**a**) Ultraviolet-visible light (UV-Vis) diffuse reflectance spectrum of Bi_5_O_7_I; (**b**) UV-Vis diffuse reflectance spectrum of Bi_5_O_7_I/Mn_x_Zn_1−x_Fe_2_O_4_. The (*ahv*)^1/2^−*hv* curve is shown in the inset image.

The UV-Vis DRS and the (*ahv*)^1/2^−*hv* curve are shown in Figure 7. The largest absorption wavelengths of Bi_5_O_7_I and Bi_5_O_7_I/Mn_x_Zn_1−x_Fe_2_O_4_ were 470 nm and 600 nm, respectively, illustrating that both could absorb ultraviolet and visible light, and that adding Mn_x_Zn_1−x_Fe_2_O_4_ extended the range of absorption light. Moreover, the band-gap energy of the samples could be obtained using Equation (1) [22].

(1) ahν=A(hν−Eg)n2，

where *α*, *h*, *v*, and *E*_g_ are the absorption coefficient, Planck constant, light frequency, and bandgap width, respectively. *A* is a constant, and *n* depends on the transition type of the semiconductor optical carriers (direct transition, *n* = 1; indirect transition, *n* = 4). According to the plots of (*ahv*)^1/2^−*hv*, the band-gap energies of Bi_5_O_7_I and Bi_5_O_7_I/Mn_x_Zn_1−x_Fe_2_O_4_ were determined to be 3.27 eV and 3.22 eV, respectively.

**Figure 8:**
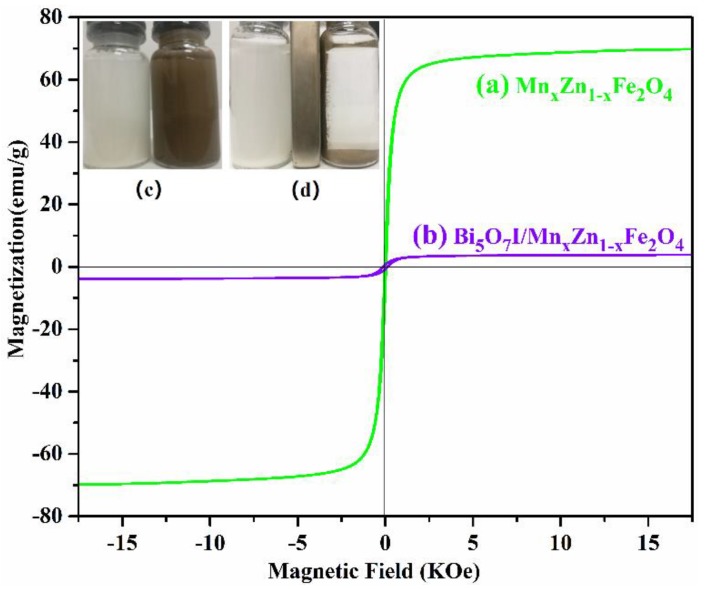
(**a**) The magnetic hysteresis loop of Mn_x_Zn_1−x_Fe_2_O_4_; (**b**) the magnetic hysteresis loop of Bi_5_O_7_I/Mn_x_Zn_1−x_Fe_2_O_4_. In the inset images, (**c**) the suspension of Bi_5_O_7_I and Bi_5_O_7_I/Mn_x_Zn_1−x_Fe_2_O_4_, and (**d**) the suspension of Bi_5_O_7_I and Bi_5_O_7_I/Mn_x_Zn_1−x_Fe_2_O_4_ are shown under the magnetic field.

In general, the magnetic performance of catalysts determines the recovery efficiency. Therefore, the magnetic hysteresis loops of the samples were measured. Figure 8b depicts that the saturation magnetization (Ms), coercivity (Hc), and residual magnetization (Mr) of Bi_5_O_7_I/Mn_x_Zn_1−x_Fe_2_O_4_ were 3.9 emu/g, 126.6 Oe, and 0.7 emu/g, respectively. Compared with Mn_x_Zn_1−x_Fe_2_O_4_, Ms declined because the mass ratio of magnetic materials in the photocatalyst was only 10%. It is worth noting that Bi_5_O_7_I/Mn_x_Zn_1−x_Fe_2_O_4_ is easy to magnetize or demagnetize, and its hysteresis loss was small in the alternating magnetic field. Figure 8d shows that the particles of Bi_5_O_7_I/Mn_x_Zn_1−x_Fe_2_O_4_ in the suspension (the right bottle) moved to the magnet rapidly when a magnet was placed close to the bottle, and the suspension became clear after 4 min. However, the suspension containing Bi_5_O_7_I (the left bottle) did not show any obvious change under the same conditions. From the above analysis, it was determined that the composite photocatalyst has great magnetic separation capabilities.

### 3.3. Photocatalytic Activity

**Figure 9:**
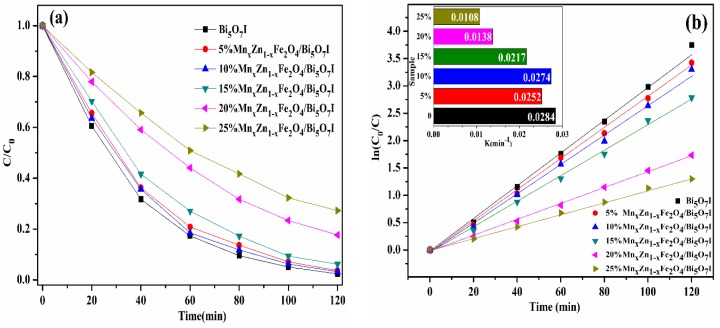
(**a**) Photocatalytic degradation and (**b**) kinetic linear simulation curves of Rhodamine B (RhB) over Bi_5_O_7_I and Bi_5_O_7_I/Mn_x_Zn_1−x_Fe_2_O_4_.

The photocatalytic activity of the as-prepared samples was investigated through photocatalytic degradation experiments of RhB. From Figure 9a, pure Bi_5_O_7_I presented the highest photocatalytic activity, whereby 97.6% of RhB was degraded within 120 min. The degradation properties of 5%, 10%, 15%, 20%, and 25% Mn_x_Zn_1−x_Fe_2_O_4_/Bi_5_O_7_I were 96.3%, 96.7%, 93.8%, 82.3%, and 72.7%, respectively. In addition, the first-order kinetics model given by equation ln(*C*_0_/*C*) = *kt* was applied to quantitatively understand the reaction kinetics, where *C*_0_ (mg·L^−1^) is the initial concentration of RhB solution, *C* (mg·L^−1^) is the concentration in aqueous solution at time *t*, and *k* (min^−1^) is the apparent first-order kinetic constant [36]. The degradation constants (in Figure 9b) were calculated to be 0.0284, 0.0252, 0.0274, 0.0217, 0.0138, and 0.0108 min^−1^ for pure Bi_5_O_7_I, and 5%, 10%, 15%, 20%, and 25% Mn_x_Zn_1−x_Fe_2_O_4_/Bi_5_O_7_I samples, respectively. Compared with Bi_5_O_7_I, the photocatalytic activity of the compounds declined for two reasons. On one hand, Mn_x_Zn_1−x_Fe_2_O_4_ became the recombination center of the photogenerated electron (e^−^) and hole (h^+^), which reduced the lifetime of the photogenerated carriers. On the other hand, adding Mn_x_Zn_1−x_Fe_2_O_4_ decreased the amount of catalyst in the compound. 

### 3.4. Stability and Recycling Ability

**Figure 10:**
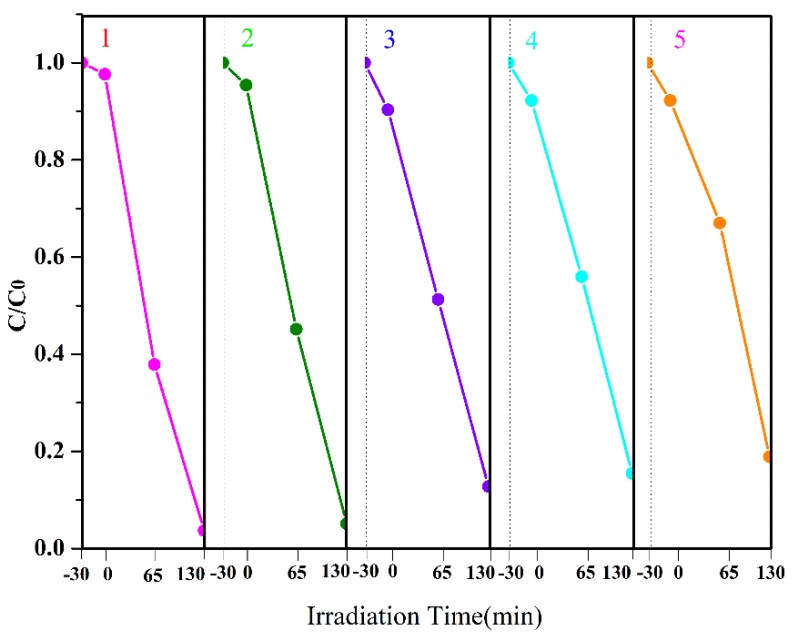
Degradation rate of RhB on Bi_5_O_7_I/Mn_x_Zn_1−x_Fe_2_O_4_ after being recycled.

The stability and the recycling ability of Bi_5_O_7_I/Mn_x_Zn_1−x_Fe_2_O_4_ were studied through recycling experiments. After each reaction, the photocatalyst was separated by an external magnet and then washed with deionized water before being dried at 80 °C for 3 h. The recycling experiments show that the average recovery rate was 90.1%. Figure 10 shows that the degradation rate was still more than 81.1% after five reuses. The experimental results indicate that the composite magnetic photocatalyst can be reused several times with excellent stability.

### 3.5. Photocatalytic Mechanism

There are a few reasons why the composite magnetic photocatalyst has good photocatalytic activity. Firstly, the existence of a porous structure is helpful for improving the transfer efficiency of the photogenerated electron and hole. In this structure, the distance from where photogenerated charge is generated to the semiconductor surface is shortened, which can effectively reduce the recombination of the photogenerated electron and hole [37].

**Figure 11:**
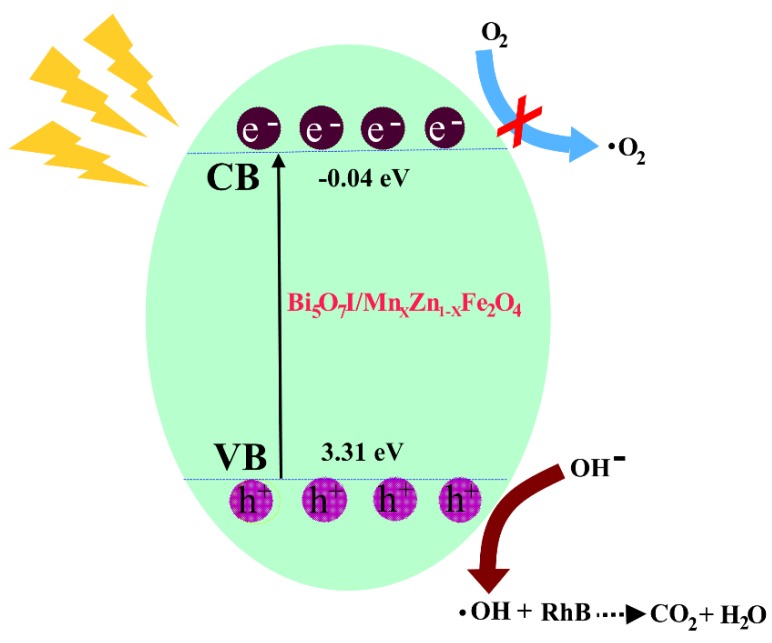
Schematic of the possible reaction mechanism of the photocatalytic procedure.

Secondly, the band structure of the photocatalyst is beneficial for producing a hydroxyl radical (•OH). Figure 11 illustrates the proposed mechanism for the photocatalytic activity of Bi_5_O_7_I/Mn_x_Zn_1−x_Fe_2_O_4_ with RhB. As is known, •OH is the major active substance in the photocatalytic degradation of organic pollutants [38]. The adsorbed water is oxidized to •OH by a hole when the valence band top has more positive redox potential than that of •OH/H_2_O (+2.27 eV). The position of the conduction band bottom (*E_CB_*) can be obtained using Equation (2) [39].

(2) $$E_{CB}=X-E^{C}-0.5E_{g},$$

where *X* is the absolute electronegativity of the semiconductor oxide, *E^C^* is the potential energy of the free electron in a standard hydrogen electrode (~4.5 eV), and *E_g_* is the band gap of the semiconductor oxide. The position of the conduction band bottom for the photocatalyst was determined to be 0.04 eV. Therefore, the position of the valence band top was 3.31 eV, which is sufficient to turn OH^−^ into •OH through oxidation.

## 4. Conclusions

The composite magnetic photocatalyst Bi_5_O_7_I/Mn_x_Zn_1−x_Fe_2_O_4_ was prepared using a hydrothermal-roasting method. This is convenient for mass production in the future because of its simple process and low cost. According to the analysis results of XRD, FTIR, XPS, SEM, and TEM, Bi_5_O_7_I and Mn_x_Zn_1−x_Fe_2_O_4_ were successfully combined. Bi_5_O_7_I/Mn_x_Zn_1−x_Fe_2_O_4_ is a mesoporous material, able to absorb ultraviolet and visible light. Meanwhile, Bi_5_O_7_I/Mn_x_Zn_1−x_Fe_2_O_4_ is a soft magnetic material with great magnetic induction intensity. The photocatalytic degradation and recycling experiments revealed that Bi_5_O_7_I/Mn_x_Zn_1−x_Fe_2_O_4_ has good photocatalytic activity and stability.
